# Clinical Remarks in the Buffalo Hospital of the Sisters of Charity

**Published:** 1874-12

**Authors:** Julius F. Miner


ART. IV.—*Clinical Remarks upon Surgical Cases occurring at the Buffalo Hospital of the Sisters of Charity*, by Professor Julius F. Miner, M. D. Reported by W. W. Miner, M. D.

Case VIII. *Dislocation of the Humerus.*— C. W. L——, aged sixty, a blacksmith by trade, received an injury on the fourteenth of August, which rendered his right arm useless. He came to this city and into my care last week. On Saturday last, the twenty-fourth of October, I attempted reduction of his dislocated shoulder, for such I found the trouble to be. It was then ten weeks since the dislocation occurred, and it was accordingly an unpromising case. With the assistance of Drs. Daggett, Sloan and others, and after considerable effort, I was able to accomplish reduction. He is now able to move the arm considerably, which could not then be done. There appears to be a deficiency in the rotundity presented by the shoulder, the acromion process seems more prominent than is natural, but it is slight in degree, and it is probable that an inflammatory deposit within the capsule of the joint and the atrophied condition of the muscles are the cause of this. Reduction was accomplished with the aid of chloroform, the heel in the axilla, traction by several persons in the line of the arm, and traction outwards by means of a towel passed around the head of the humerus, while forcible manipulation of the joint was carefully made. It is remarkable that no serious nerve trouble has been present in the case. A month or more after reduction the man was found to be able to use his arm in eating and in common duties; the shape of the joint had improved and was nearly perfect.

Case IX. *Talipes Valgus.*—This child is one which has been operated on some time since, I am told, for the relief of the affection which you notice is still present in a degree in its foot. It presents the appearance of an outwardly inclined club-foot. The position of the foot and ankle here is one which may be produced by various causes; the fascia connected with the parts may be contracted so as to need extensive division in remedying the obliquity of the foot; the tendons of the peroneal muscles may be short and require division; the bones of the foot and ankle may be so deformed as to favor abnormal position, and these may in some instances be moulded into suitable shape and position. In the present instance none of these causes seem especially operative. When the child stands on the foot, it places it in a very everted position, but the foot is not firmly held in that position when the weight of the body does not rest upon it. In fact, when walking there seems to be a lack of firmness in the position assumed by the ankle. What is the trouble here? There is a lack of restraint in the lateral directions rather than any unnatural fixity or tension. The tendo achillis is too short, though this may not be noticeable until I hold the foot squarely, and attempt to flex it to the extent naturally required in walking; it scarcely comes to a right angle with the leg, cannot be flexed beyond this degree. The tendo achillis is too short to allow the natural movement of the ankle in walking, and in order to overcome this disability, the child everts the foot, and you see how well the projecting of the toe is avoided in this way. This is not a position of eversion from causes that are constantly operative, it is an eversion assumed as compensatory to the not greatly noticeable degree of equinus that exists, and is permanent. To remedy the trouble I simply divide the tendo achillis, and the result is entirely satisfactory. There is some degree of atrophy of the muscles of the leg here; of this and other points connected with the subject of club-foot I will say more in future. The foot is fastened in proper position with adhesive straps, over which a roller bandage is applied. A shoe proper for this retention may hereafter be applied if desirable.

Case X. *Chronic Ulcer.*—Your reputation as a physician or surgeon will depend as largely perhaps on your management of such cases as this, as of any other cases you have. Not one thousandth part of those who have ulcers, come to hospitals for treatment, still in all hospitals, county houses or asylums, you will find these cases more or less numerous. They will very frequently come to you. Almost all ulcers not specific will get well if the patients are put to bed. If specific they require their appropriate treatment, if dependent upon varicose veins these must be attended to. If dependent upon an impoverished condition of the system, as is generally the case, it is proper to make use of tonic remedies, hygienic means and general constitutional treatment. If the edges and surface of the sore is callous and of low vitality, pare it well with the knife. If the surface merely is of indolent character, caustic applications may be used. Poultices may suffice for the removal of inactive tissues. Stimulating ointments are of service. A firm bandage carefully and evenly applied, is often valuable. The means that may be made of avail are very numerous, and should be successful if thoroughly and persistently adopted. We are able to furnish a covering of skin for healthy surfaces of large extent, now, by means of the operation called "transplantation." This operation though made use of by Prof. Frank H. Hamilton, in this hospital in 1854, and previously advocated as a plastic operation by him, was practically introduced as now performed by M. Reverdin in one of the hospitals of Paris. A few weeks after the announcement of the method and success of Reverdin, the operation was introduced at the clinic here. It consists in taking grafts of healthy skin of minute size and implanting them upon the granulating surface of an ulcer which it is desired shall be covered with epidermis. A few normal centers of proliferation will thus speedily cover an extent of raw surface that in old times would never have been healed.

Case XI. *Extraction of Cataract.*--M—— R——, aged 72, born in France, has cataract in both eyes, which has progressed to such an extent that he is now obliged to give up his usual occupation, and seeks relief by the operation for removal of the lens. Two grand distinctions in a cataractous lens are those of "hard" and "soft." If the case is one of soft cataract there is no need of extraction of the lens. If the lens substance is brought into contact with the aqueous humour it is absorbed, and will disappear in the course of five or six weeks, although the opacity may have been of great degree. The operation called "couching" was formerly very much practiced. All surgeons were in the habit of depressing cataracts. This operation consists in introducing an instrument into the eye and with it depressing the cataractous lens from its position in the line of vision, displacing it more or less into the vitreous body, and out of the way of the rays of light entering the pupil. This operation would no doubt be the best one to be practiced by inexperienced surgeons now. The objection to the operation is that good results at first obtained may be followed six months afterwards by destructive inflammation of the eye. I have seen excellent results from couching; have a case in mind in which I performed this operation twenty years since, and vision is now very satisfactory. The operation of couching is no longer considered proper by ophthalmologists. It leaves a hard nucleus within the eye that sometime may be the cause of trouble. Removal of the lens is the only proper procedure with hard cataracts. This may be effected in various ways, suction has been made use of for the removal of the lens; in this method only a small opening in the wall of the globe is required, but the method is practicable only when the lens is soft. The most common way is by making a suitable incision, and then removing the lens entire if possible, or in successive portions, by mechanical means. The matter of the incision in the operation for extraction of cataract is one upon which a great deal has been said, and which has divided ophthalmic surgeons to a great extent. I cannot say to-day which incision has received the most general sanction. In the present case I enter Von Graefe's knife in the lower third of the cornea at the sclero-corneal junction immediately in front of the iris, and cut downwards and outwards nearly in the direction of the axis of the globe. Having ruptured the capsule sufficiently with a suitable instrument, I accomplish removal of the lens with a needle or scoop. After closing the lids and a period of rest, the eye is again examined to see if any unremoved portions of lens are present; the corneal wound is carefully examined, and if any portion of iris is engaged in the wound it is replaced within, or friction over the closed lids causes contraction of the iris, and thus removal is accomplished, so that the corneal wound is left perfectly clean and properly apposed. A graduated compress is applied, and the patient should now be kept in bed, or his eyes excluded from light, for ten days or more. I have more often made incision upwards than downwards; used to be careful to have the incision small for fear of prolapse of the vitreous, which I no longer fear. Iridectomy is recommended by many, and generally or always performed by some; it causes deformity, and where it is not necessitated I am not in the habit of making it. Non-union of the corneal wound is a source of failure in these operations, but as a rule operations for the extraction of cataract are successful. Good vision generally results. Distant objects can be seen without glasses, while for near objects a suitable convex lens is required. The patient left the hospital at the end of about three weeks, at which time he was presented at clinic and was observed to have a pretty clear condition of the eye, with which he could distinguish objects readily, and which promised useful vision.

